# Attention flexibility is associated with retinal cup-to-disk ratio in patients with schizophrenia spectrum disorders

**DOI:** 10.1192/j.eurpsy.2024.601

**Published:** 2024-08-27

**Authors:** S. Jerotic, N. Maric

**Affiliations:** ^1^Faculty of Medicine, University of Belgrade; ^2^Clinic for Psychiatry, University Clinical Centre of Serbia; ^3^Institute of Mental Health, Belgrade, Serbia

## Abstract

**Introduction:**

In recent years, there has been increasing interest in the potential use of retinal imaging as a non-invasive and easily accessible tool for investigating the neurobiological underpinnings of schizophrenia. Studies have suggested that patients with schizophrenia spectrum disorders (SSD) have structural abnormalities in the retina, including changes in retinal thickness and the ratio of the retinal cup-to-disk ratio.

**Objectives:**

To investigate the relationship between retinal cup-to-disk ratio and cognitive performance in patients with SSD using a high-definition retinal imaging device – optical coherence tomography (OCT) scanner.

**Methods:**

The sample was comprised of twenty patients with SSD (F20-F29 according to ICD-10 criteria). All diagnoses were confirmed by a researcher using the Mini International Psychiatric Interview. All patients underwent complete ophthalmological examination, excluding any ocular pathology. Retinal thickness was measured in both eyes of all patients with a high-definition spectral-domain OCT device. Examined retinal parameters were: total retinal nerve fiber layer thickness (RNFL); RNFL thickness in all eye quadrants (nasal, temporal, superior, inferior); RNFL symmetry; average macular volume (MV); average macular thickness (MT); ganglion cell layer thickness (GC); average retinal cup-to-disk (C/D) ratio, vertical C/D ratio. Cognitive performance of all patients was tested using the Intra/Extradimensional Set Shift Task (IED). IED is a component of a state-of-the-art computerized battery for cognitive assessment – Cambridge Neuropsychological Automated Test Battery. IED is a measure of maintenance, shifting and flexibility of attention. Associations between retinal variables and IED measures were determined with Pearson correlation analyses.

**Results:**

Mean age of patients was 33 ± 7.5 years. Fifty five percent of the sample was male, illness duration was 6.2 ± 3.9 years. Daily dosage of chlorpromazine was 225.7 ± 108.8 mg. Retinal C/D ratio in the right eye was positively associated with IED total errors (r=0.50; p=0.02) and negatively with IED stage progression (r=-0.52, p=0.18). Likewise, vertical C/D ratio was positively associated with IED total errors (r=0.49; p=0.02) and negatively with IED stage progression (r=-0.52, p=0.18).

**Conclusions:**

Previous analyses of retinal parameters in patients with schizophrenia point towards enlargement of retinal cup-to-disk ratio, irrespective of any underlying somatic comorbidities. Our data shows worsening of attention flexibility in association with the increase of cup-to-disk ratio in patients with schizophrenia spectrum disorders. The significance of cup-to-disk retinal disturbance in schizophrenia spectrum disorders and its connection with cognitive performance should be further evaluated and supplemented with measurements of functional adaptation in these patients.

**Disclosure of Interest:**

None Declared

